# Effect of polyp regression and reduction on treatment efficacy in polypoidal choroidal vasculopathy treated with aflibercept

**DOI:** 10.1038/s41598-024-52448-y

**Published:** 2024-01-21

**Authors:** Kaori Sayanagi, Satoko Fujimoto, Chikako Hara, Yoko Fukushima, Kazuichi Maruyama, Ryo Kawasaki, Shigeru Sato, Kohji Nishida

**Affiliations:** 1 Department of Ophthalmology E7, Osaka University Graduate School of Medicine, 2-2 Yamadaoka, Suita, 565-0871 Japan; 2 Integrated Frontier Research for Medical Science Division, Institute for Open and Transdisciplinary Research Initiatives, Osaka University, Suita, Japan

**Keywords:** Retinal diseases, Outcomes research

## Abstract

Intravitreal injection of aflibercept (IVA) has successfully treated polypoidal choroidal vasculopathy (PCV), and polyp morphology is an important indicator of treatment efficacy. However, many studies have not reported the presence or absence of polyp regression and treatment outcomes, and few studies have reported polyp reduction and treatment outcomes in cases with residual polyps. We retrospectively measured the polyp area on indocyanine green angiography images before and after the IVA loading phase and investigated the regression and reduction of polyps and treatment outcomes of 81 eyes with PCV treated with IVA. We investigated the relationship between the presence or absence of complete regression of polyps and the percentage change in the polyp area and treatment outcomes. Eyes with complete polyp regression had significantly better visual acuity improvements compared with baseline at 12 months (*P* = 0.0108), fewer treatments (*P* = 0.0024), fewer recurrences during 12-months follow-up (*P* = 0.0010), and more “dry maculas” at 3 months (*P* = 0.0048) than eyes in which polyp regression did not occur. A significant correlation was seen only between the percentage of polyp regression and visual acuity at 3 months (*P* = 0.0395). Regarding IVA therapy for PCV, the presence or absence of complete polyp regression at the end of the loading phase affected the treatment outcome, whereas the degree of polyp reduction in cases of residual polyps had no effect.

## Introduction

Age-related macular degeneration (AMD) is a major cause of legal blindness worldwide and in developed countries^1^. Polypoidal choroidal vasculopathy (PCV), a subtype of AMD, has a high incidence in Asia^2^. The diagnosis of PCV was determined originally based on the presence of polypoidal lesions and abnormal branching vascular networks (BVNs) on indocyanine green angiography (ICGA) images, although recent reports have indicated that noninvasive imaging, i.e., optical coherence tomography (OCT) and OCT angiography (OCTA), are used to visualize PCV^3,4^. The mainstay of PCV treatment is anti-vascular endothelial growth factor (VEGF) monotherapy or in combination with photodynamic therapy (PDT) and long-term favorable visual acuity (VA) has been reported with both therapies^5–11^. The efficacy of PCV treatment is evaluated based on the VA, number of treatments, exudative changes, and polyp regression rate. Complete polyp regression rates in various clinical trials have been reported ranging from 26 to 29% for ranibizumab (Lucentis®; Novartis Pharma AG, Basel, Switzerland) monotherapy, 39–77% for aflibercept (Eylea®; Bayer HealthCare, Berlin, Germany) monotherapy, 79% for brolucizumab (Beovu®, Novartis, Basel, Switzerland) monotherapy, 77% for PDT combined with aflibercept, and 20–78% for PDT combined with ranibizumab^5–9,12–17^. Complete polyp regression is considered related to the incidence of long-term subretinal hemorrhage, the number of treatments, and the recurrence rate, although the relationship with the VA is uncertain^12,18–21^. Although polyp regression rate, number of polyps, and lesion area including BVNs are used as indicators of PCV pathological change used in the discussion of treatment outcomes^12,18–24^, these indicators do not seem to adequately assess polyps when polyp shrinkage without resolution. The purpose of this study was both to investigate whether polyp reduction or shrinkage affects the outcomes of patients with residual polyps after the loading phase of anti-VEGF therapy and to explore whether the percentage change of the polyp area could be a novel prognostic factor, which has not been reported in previous studies. Therefore, we measured the polyp area before and after intravitreal injection of aflibercept (IVA) and used the percentage change in the polyp area to examine the relationship between polyp regression, shrinkage, and treatment outcomes.

## Results

The baseline and 12-month characteristics of the 81 cases are summarized in Table 1.

**Table 1:** Demographics, baseline characteristics, BCVA at baseline.

Clinical parameters	Polyp regression (+) (n = 48)	Polyp regression (−) (n = 33)	*P* value
Gender (male/female)	33/15	24/9	0.8064
Age (years)	74.5 ± 7.7	72.5 ± 5.5	0.3034
LogMAR BCVA at baseline	0.32 ± 0.32	0.23 ± 0.41	0.0405*
Baseline polyp area (mm^2^)	0.14 ± 0.15	0.25 ± 0.23	0.0013*
CRT (microns)	385.8 ± 176.5	340.4 ± 177.9	0.0993

*P < 0.05.

**Figure 1:**
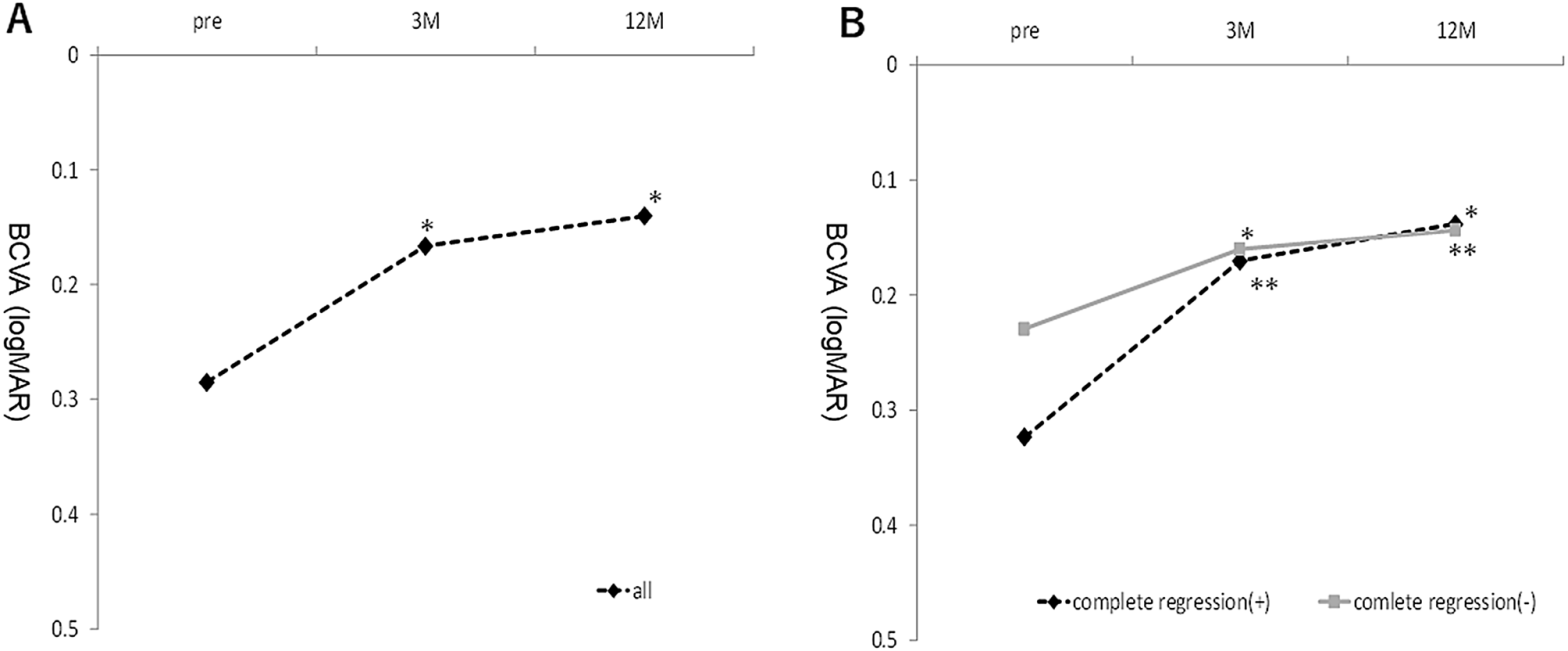
(**A**) The mean logMAR BCVA in all 81 eyes with PCV treated with IVA injections over a 12-month period. (**B**) The mean logMAR BCVA in eyes with and without complete regression of polyps. *P < 0.05 versus baseline. **P < 0.05 versus baseline. *M* months.

**Figure 2:**
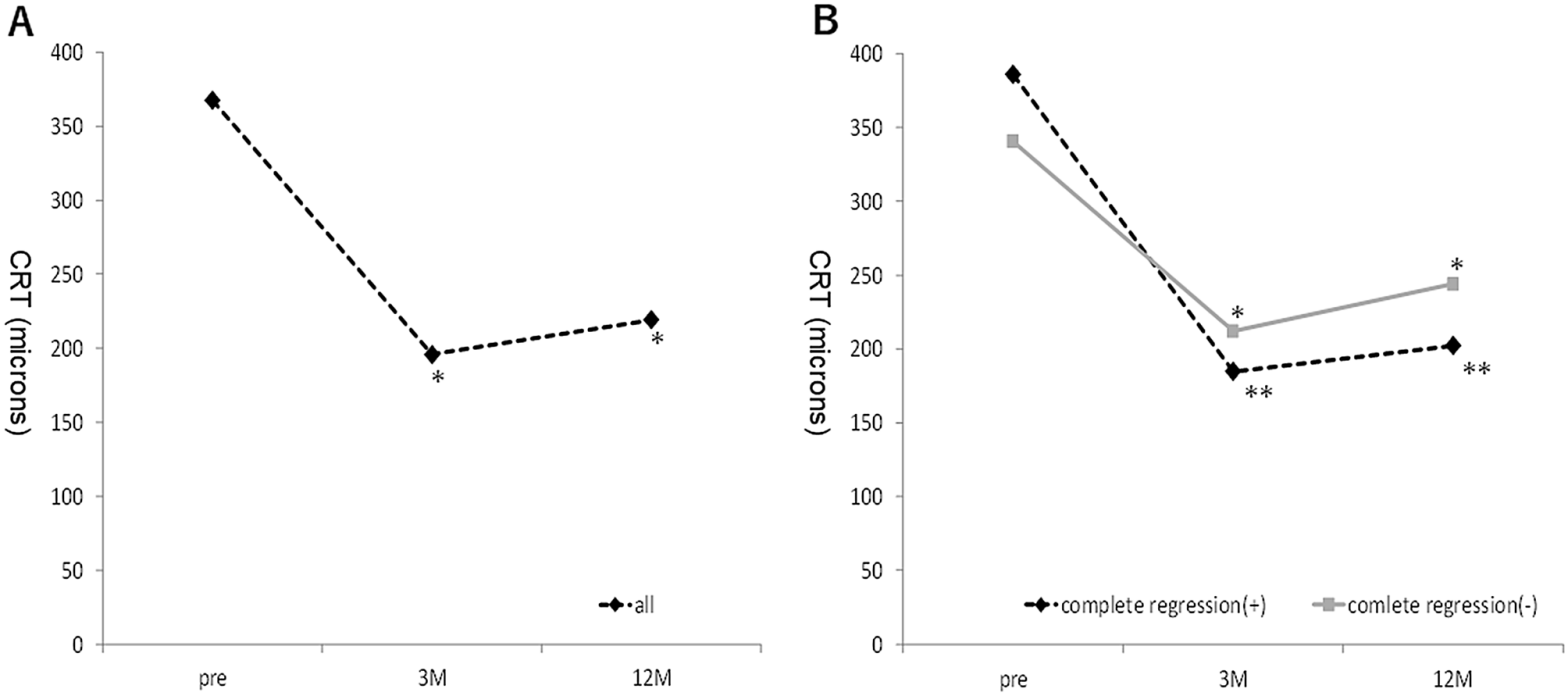
(**A**) The mean CRT in all 81 eyes with PCV treated with IVA injections over a 12-month period. (**B**) The mean CRT in eyes with and without complete regression of polyps. *P < 0.05 versus baseline. **P < 0.05 versus baseline. *M* months.

The mean patient age was 73.7 ± 6.9 years, and 57 cases (70%) were men. During the 12-month follow-up, the mean number of treatments was 5.7 ± 2.6. All patients were treated with IVA until 12 months except one who switched to intravitreal ranibizumab (IVR) during follow-up. The mean total polyp area before treatment was 0.18 ± 0.19 mm^2^, which decreased significantly to 0.04 ± 0.09 mm^2^ 3 months after treatment (*P* < 0.0001). The mean percentage of polyp regression was 83.2% ± 26.5% at 3 months. Of the 81 cases of PCV, 48 (59%) had complete polyp resolution and 33 (41%) had residual polyps. Of the latter, the polyp area increased from pre-treatment in only one case. Overall, the best-corrected VA (BCVA) improved significantly from 0.29 ± 0.36 logarithm of the minimum angle of resolution (logMAR) unit at baseline to 0.17 ± 0.31 logMAR unit at 3 months (*P* < 0.05) and 0.14 ± 0.32 logMAR unit at 12 months (*P* < 0.05). In the patients with complete polyp regression, the BCVA was 0.32 ± 0.32 unit at baseline, 0.17 ± 0.26 unit at 3 months, and 0.14 ± 0.28 unit at 12 months, with significant differences between the pre-treatment and post-treatment BCVA (*P* < 0.05 for both comparisons at 3 and 12 months, respectively). In the patients with residual polyps, the BCVA was 0.23 ± 0.41 unit at baseline, 0.16 ± 0.38 unit at 3 months, and 0.14 ± 0.37 unit at 12 months, with significant differences between the pre-treatment and post-treatment BCVA (*P* < 0.05 for both comparisons at 3 and 12 months, respectively) (Fig. 1). Overall, the central retinal thickness (CRT) improved significantly from 367.3 ± 177.4 microns at baseline to 195.9 ± 61.8 microns at 3 months (*P* < 0.05) and 219.2 ± 110.2 microns at 12 months (*P* < 0.05). In the patients with complete polyp regression, the CRT was 385.8 ± 176.5 microns at baseline, 184.9 ± 50.3 microns at 3 months, and 202.3 ± 67.4 microns at 12 months, with significant differences between the pre-treatment and post-treatment CRT (P < 0.05 for both comparisons at 3 and 12 months, respectively). In the patients with residual polyps, the CRT was 340.4 ± 177.9 microns at baseline, 211.9 ± 73.5 microns at 3 months, and 219.2 ± 110.2 microns at 12 months, with significant differences between the pre-treatment and post-treatment CRT (*P* < 0.05 for both comparisons at 3 and 12 months, respectively) (Fig. 2).

### Relationship between complete polyp regression and treatment outcome

Patients with complete polyp regression (n = 48; 59%) had a significantly better pretreatment BCVA (*P* = 0.0405) and significantly smaller polyp area (*P* = 0.0013) than the patients without complete polyp regression (n = 33; 41%), although there was no difference in the sex ratio (*P* = 0.8064), age (*P* = 0.3034), or CRT (*P* = 0.0993). Following treatment, patients with complete polyp regression had significantly better BCVA changes from baseline at 12 months (*P* = 0.0108, Bonferroni corrected *P* value), fewer treatments (*P* = 0.0024), fewer recurrences during the 12-month follow-up after the loading phase (*P* = 0.0010), and more dry maculas at 3 months (*P* = 0.0048, Bonferroni corrected *P* value), than those without complete polyp regression, although there was no significant difference in the BCVAs at the 3 months (*P* = 0.6135, Bonferroni corrected *P* value) and 12 months (*P* = 1.5309, Bonferroni corrected* P* value), BCVA changes from baseline at 3 months (*P* = 0.0562, Bonferroni corrected* P* value), dry maculas at 12 months (*P* = 0.4773, Bonferroni corrected *P* value), and CRTs at 3 months (*P* = 0.1827, Bonferroni corrected P value) and 12 months (*P* = 0.1203, Bonferroni corrected *P* value) (Table 2).

**Table 2:** Comparison of treatment outcomes between eyes without and without complete polyp regression.

Clinical parameters	Polyp regression (+) (n = 48)	Polyp regression (−) (n = 33)	*P* value	Bonferroni corrected* P* value
LogMAR BCVA at 3 months	0.17 ± 0.26	0.16 ± 0.38	0.2045	0.6135
LogMAR BCVA at 12 months	0.14 ± 0.28	0.14 ± 0.37	0.5103	1.5309
BCVA change from baseline at 3 months	− 0.15 ± 0.19	− 0.07 ± 0.15	0.0281	0.0562
BCVA change from baseline at 12 months	− 0.19 ± 0.20	− 0.09 ± 0.16	0.0054	0.0108*
CRT at 3 months (microns)	184.9 ± 50.3	211.9 ± 73.5	0.0609	0.1827
CRT at 12 months (microns)	202.3 ± 67.4	243.8 ± 150.4	0.0401	0.1203
Dry macula at 3 months, n (%)	44 (91.7%)	20 (60.6%)	0.0016	0.0048*
Dry macula at 12 months, n (%)	28 (58.3%)	14 (42.4%)	0.1591	0.4773
Total number of injections, n	5.0 ± 2.4	6.7 ± 2.7	0.0024*	
Recurrence after loading phase, n (%)	27 (56.3%)	30 (90.9%)	0.0010*	

*P < 0.05.

### Relationship between the percentage change in polyp area and treatment outcomes

A significant correlation was seen between the percentage change in polyp area and BCVA at 3 months (*P* = 0.0395) but not with the BCVA at 12 months (*P* = 0.1889), BCVA changes from baseline at 3 months (*P* = 0.7532) and 12 months (*P* = 0.1497), total number of treatments (*P* = 0.7738), CRT at 3 months (*P* = 0.6927) and 12 months (*P* = 0.9357), dry maculas at 3 months (*P* = 0.6903) and 12 months (*P* = 0.3106), or presence or absence of recurrence during the follow-up period (*P* = 0.1606) (Table 3).

**Table 3:** Univariate regression analyses of the percentage of polyp regression and treatment outcome.

Clinical parameters	B	SE	Lower bound of 95% CI	Upper bound of 95% CI	*P* value
LogMAR BCVA at 3 months	0.0051	0.0024	0.0003	0.0099	0.0395*
LogMAR BCVA at 12 months	0.0033	0.0024	− 0.0017	0.0082	0.1889
BCVA change from baseline at 3 months	0.0003	0.0001	− 0.0017	0.0023	0.7532
BCVA change from baseline at 12 months	− 0.0015	0.0010	− 0.0035	0.0006	0.1497
Total number of injections	− 0.0051	0.0177	− 0.0413	0.0310	0.7738
CRT at 3 months	− 0.1959	0.4909	− 1.1971	0.8054	0.6927
CRT at 12 months	− 0.0819	1.0071	− 2.1358	1.9721	0.9357

Clinical parameters	Odds ratio	Lower bound of 95% CI	Upper bound of 95% CI	*P* value	
Recurrence after loading phase	1.0558	0.9941	1.1678	0.1606	
Dry macula at 3 months	0.9946	0.9687	1.0213	0.6903	
Dry macula at 12 months	0.9858	0.9567	1.0124	0.3106	

*M* months, *SE* standard error, *CI* confidence interval.

*P < 0.05.

## Discussion

In the current study, we measured the polyp areas in patients with PCV before and after anti-VEGF therapy and examined the relationship between polyp regression and the percentage change in the polyp area and treatment outcomes. The results showed that cases with complete regression of polyps required significantly fewer treatments and had fewer recurrences during the follow-up period, a better change in the BCVA from baseline at 12 months and a higher rate of dry maculas at 3 months compared to cases without complete polyp regression. However, no significant differences between the two groups were seen in the BCVAs at 3 and 12 months, BCVA changes from baseline at 3 months, dry macula rate at 12 months, or CRT at 3 months and 12 months**.** Moreover, the percentage change in the polyp area was not associated significantly with either the BCVA at 12 months, BCVA change from baseline, CRT, or dry macula rates at 3 and 12 months, number of treatments, or recurrence during the follow-up period, although it was significantly associated with the BCVA at 3 months.

In this study, all 81 cases of PCV were treated with anti-VEGF therapy, i.e., aflibercept, except for one patient who switched to ranibizumab during the treatment, and 48 of the 81 patients (59%) achieved complete regression of polyps. This is comparable to the previously reported rates of complete regression of polyps with IVA for PCV^6,7,14,15,17^. Morizane-Hosokawa et al. evaluated polyp regression and treatment outcomes in patients with PCV who underwent IVA and reported significantly fewer treatments, significantly fewer recurrences, and longer treatment intervals in the group that achieved complete polyp regression compared to the group that did not^19^. Likewise, Matsumoto et al. reported that patients with PCV treated with brolucizumab and achieved complete polyp regression had significantly fewer treatments and a longer treatment interval than those who did not achieve it^20^. However, the reports on the BCVA were divided regarding whether or not there was a significant correlation between complete polyp regression and BCVA. In patients with PCV treated with IVA, Morizane-Hosokawa et al. found that complete regression of polyps at 3 months was not significantly associated with the BCVA improvement at 2 years^19^, while Chaikitmongkol et al. reported that patients with complete polyp regression had significantly better BCVA improvement than those who did not^21^. In the current report, patients who achieved complete regression of polyps had significantly better changes in the BCVA at12 months, fewer number of treatment and fewer recurrence during the 12-months follow-up period than those who did not. Based on the above findings, the outcome of IVA treatment for PCV for at least 1 year was better in cases in which complete regression of polyps was achieved. Hence, instead of IVA, a reasonable initial treatment option to consider would be a treatment with a higher polyp regression rate, such as brolucizumab or a PDT combination. However, some studies have reported long-term preservation of BCVA even with ranibizumab, which has a low polyp regression rate^10^, and brolucizumab can cause intraocular inflammation and PDT can result in impaired long-term VA^8,10,25,26^. Therefore, a direct comparison of the therapeutic outcomes of these treatment methods would be necessary to determine the optimal initial treatment.

Previous reports have investigated the effects of the number of polyps and PCV lesion area associated with residual polyps on treatment prognosis^22–24^. The number of polyps, however, does not reflect the polyp shrinkage, which may be associated with reduced exudation and better treatment outcomes. Similarly, the PCV lesion area does not represent polyp changes, especially in cases with a large BVN lesion. Therefore, in the current study, we measured only the polyp area and calculated the percentage change in that area to examine the relationship between the polyp lesions and treatment outcome in cases with residual polyps. Ogasawara et al. reported that the PCV lesion area and polyp number did not affect the 12-month VA in patients with PCV treated with IVA^22^, and Mori et al. reported that the polyp number, location, and type did not affect the recurrences during follow-up or VA at 24 months in patients with PCV treated with IVR^24^. However, Akagi‑Kurashige et al. reported that the presence or absence of polyps, PCV lesion size, and cystoid macular edema affected the 5-year maintenance of vision in PCV cases.^23^ These reports included cases of complete polyp regression, and as described previously, the number of polyps and PCV lesion area may not adequately evaluate the cases with polyp shrinkage. In the current study, since only cases with residual polyps were analyzed and the area of polyps only was measured, a more accurate evaluation of the polyp changes may have been achieved. Our results also showed that the change in the polyp area was correlated with the BCVA at 3 months but not at 12 months, BCVA changes from 3 to 12 months, CRT, dry macula rate, or number of injections. Additional treatment might not be warranted to reduce or shrink polyps in patients with residual polyps after the loading phase of anti-VEGF therapy. Since this was a short-term, single-center study, further investigation is required to determine the desirability of additional treatment.

In the current study, polyps were observed using ICGA at 3 months after induction of anti-VEGF therapy. Using ICGA, Chaikitmongkol et al. observed patients with PCV who underwent IVA therapy for 1 year and reported that polyp regression occurred in 55% of cases at 2 months and 77% at 6 months after the onset of treatment^21^. Conversely, Hara et al. and Yamamoto et al. reported that even when the polyps regressed completely, a low percent of polyps recurred during the first year of treatment^14,17^. Further studies are needed to determine whether this timing is appropriate and for how long the presence or absence of polyp regression at 3 months affects the outcomes. Recently, Bo et al. reported that recurrence under treatment for PCV was associated significantly with progression of BVN and polyp lesions observed by OCT angiography^27^. In the future, combined evaluation using non-invasive OCT and OCT angiography and ICGA will be increasingly necessary^4,27–29^.

The current study had several limitations including its retrospective design and it was performed in one center with a small number of cases. It would have been preferable to observe changes in polyp lesions sequentially through ICGA images obtained until 12 months. A comparison with the morphology of BVN and polyps on OCT angiography images, which we did not perform in this study, is another issue to be explored. Although this study analyzed OCT images of the fovea, it would be more accurate to evaluate macular fluid if scans of the perifoveal area also were analyzed.

In summary, we investigated the association between polyp morphology and treatment outcome in patients with PCV who underwent IVA and examined the outcomes of patients with residual polyps by measuring the polyp area before and after the loading phase. The results showed that the presence or absence of complete regression of polyps at the end of the loading phase may be helpful for predicting the therapeutic outcome and that the reduction or shrinkage of polyps does not affect the therapeutic outcome.

## Methods

This was a retrospective, observational study based on the medical records of patients treated at Osaka University Hospital Osaka, Japan. A total of 81 eyes of 81 patients with PCV were included. No patients had been treated previously for PCV and began treatment with IVA starting after January 2013, with at least 12 months of follow-up since starting treatment. The exclusion criteria included ocular turbidity such as severe cataract, severe vitreous opacity, and/or severe hemorrhage that caused blurring of the fundus and angiography images; and a history of pars plana vitrectomy. Eligible and noneligible cases were determined based on fundus photographs, OCT findings, and angiographic findings. The research adhered to the tenets of the Declaration of Helsinki. The institutional review board of Osaka University Hospital approved this retrospective study. Informed consent was obtained in the form of an opt-out option on the website.

### Clinical examinations

At each follow-up visit, the patients underwent a complete ophthalmic examination, which included measurement of the BCVA, slit-lamp biomicroscopy, dilated funduscopy, color fundus photography (TRC-50DX, Topcon Corporation, Tokyo, Japan), and OCT (swept-source OCT DRI OCT-1 Atlantis, Topcon Corporation and/or swept-source OCT DRI OCT-1 Triton, Topcon Corporation) at all visits during the 12-month follow-up. The decimal BCVA was measured using the Landolt chart and was expressed in logMAR units. Angiography was performed with fluorescein angiography and ICGA at baseline using the Heidelberg Retina Angiograph + OCT (Heidelberg, Germany) and a fundus camera (TRC-50DX, Topcon Corporation) before and 3 months after treatment.

### Diagnosis of PCV

Retina specialists (K.S., C.H., S.S., and Y.F.) diagnosed the PCV in cases with punctate hyperfluorescent spots on ICGA and at least one of the following clinical or angiographic findings: BVN, pulsatile polypoidal lesions, orange subretinal nodule, hypofluorescent halo, or associated with a massive submacular hemorrhage^3^.

### Outcome measures

The presence or absence of polyp regression on ICGA before and after the IVA loading phase was observed and the percentage change in the polyp area in cases with residual polyps was obtained and investigated in correlation with the BCVA, visual improvement, and the presence or absence of recurrence during follow-up, the dry macula rate, number of treatments, and CRT. The CRT was defined as the distance between the surface of the internal limiting membrane and the bottom of the retinal pigment epithelium (Bruch's membrane in cases with a pigment epithelium detachment) at the foveal center. CRT was measured manually using the OCT caliper function in the instrument. A dry macula was defined as the absence of fluid in the foveal center cross-section of the OCT images, either intraretinally, subretinally, or in the subretinal pigment epithelium.

### Treatment strategies

After topical anesthesia was applied, an injection of an anti-VEGF drug, aflibercept, was administered 3.5–4.0 mm posterior to the corneal limbus into the vitreous cavity using a 30- or 34-gauge needle. Prophylactic topical antibiotics were applied for 3 days after the injection. After the initial treatment, additional treatment was based on a modified treat-and-extend (TAE) regimen, which has been reported previously^30^. Briefly, three initial loading doses of intravitreal anti-VEGF therapy were administered, followed by monthly injections until a dry macula was achieved, and the TAE regimen began when the need for additional treatment was diagnosed. When the retinal exudates resolved, the interval to the next injection and follow-up period was extended by 2 weeks up to a maximum of 12 weeks. If the exudation recurred, the interval was shortened by 2 weeks to a minimum interval of 4 weeks. Follow-up was generally every 2 months in cases in which no fluid or hemorrhage was detected. The criteria for additional treatment were determined based on objective/subjective visual declines or new hemorrhage or recurrent exudative changes seen on OCT. If fluid persisted after six monthly IVAs, the patient was switched to another anti-VEGF agent (ranibizumab).

### Quantification of polypoidal lesions on ICGA

**Figure 3:**
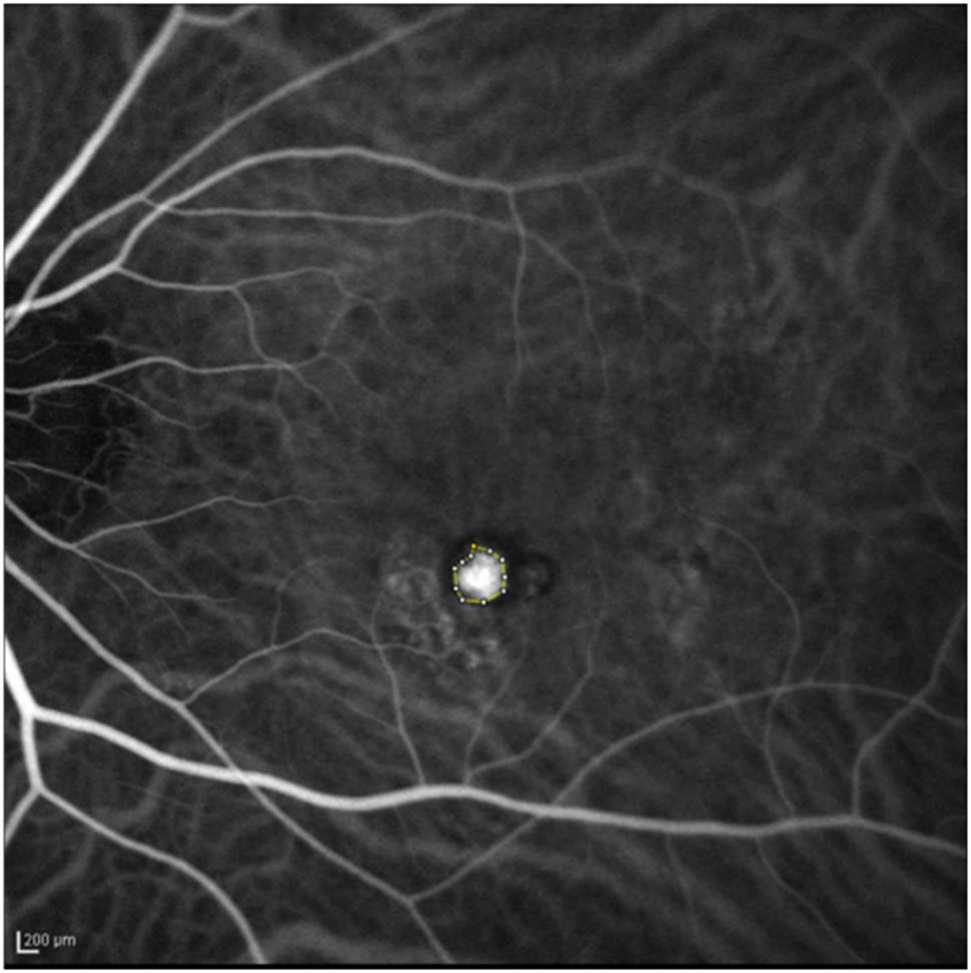
An example of measurement of the polyp area. The polyp lesion area of this image is 0.220 mm^2^.

Quantification of the polypoidal lesions was based on the ICGA findings. ICGA with an angle of view of 30° was performed for up to 20 min using the Heidelberg Retina Angiograph + OCT, and this study used images obtained around 1 min after the start of angiography^31–33^. The polyp lesions were calculated using ImageJ software (National Institutes of Health, Bethesda, MD, USA). Since pixels must be calculated and scaled when using this software, 200 microns was equivalent to 15 pixels when calculated using ImageJ software. The data were entered into the ImageJ set scale to calculate the area. The polygonal sections then were used to enclose and measure the total area of the polyp lesions. These measurements were performed twice on different days and the average of the measurements was used for statistical analysis. Figure 3 shows an example of the measurement of the polyp lesions. The percentage change in the polyp area was calculated by comparing the polyp area before treatment and 3 months after the loading phase of anti-VEGF therapy. A 100% reduction in polyp area was defined as complete regression of polyps.

### Statistical analysis

Statistical analysis was performed using JMP Pro software version 17.1.0 (SAS Institute, Cary, NC, USA). Qualitative data were compared using the Fisher’s exact test. The Wilcoxon t-test was used for nonparametric numerical data. The Bonferroni correction is used when several dependent or independent statistical tests are being performed simultaneously. Univariate linear regression analyses were performed to determine the associations of the percentage change in the polyp area and treatment outcome. One-way analysis of variance was used to compare the BCVA and CRT before and after treatment. *P* < 0.05 was considered significant.

## Data Availability

All data generated or analyzed during this study are included in this published article.
